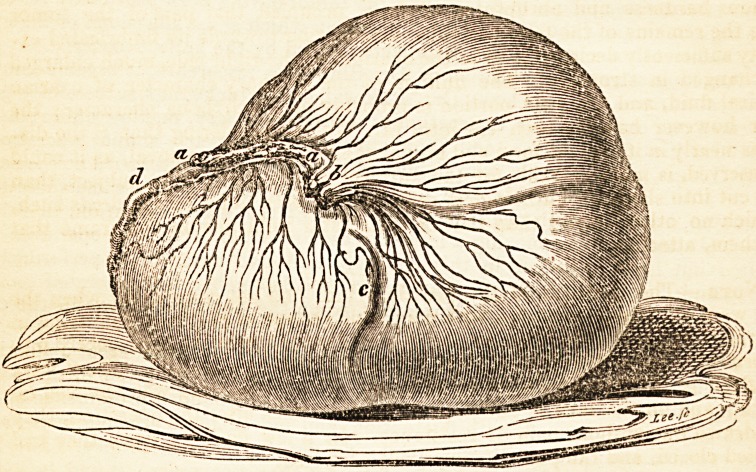# Extra-Limites

**Published:** 1843-04-01

**Authors:** 


### EXTRA-LIMITES.

### Removal of a Dropsical Ovarium, entire, by the Large Abdominal Section. By *D. Henry Walne*, Esq.

It is now about twenty years since Dr. Blundell advanced the opinion that "extirpation of the ovarian cyst in scirrhus combined with dropsy, or in simple dropsy," would, as an operation, "ultimately come into general use." He was enabled, however, to refer to two cases only, in both of which what may be termed the minor operation was performed, the incision into the abdomen being only about three inches in extent, and the tumor having been previously reduced in bulk by tapping the cyst. One of these, in which some adhesions of the cyst to the omentum existed, and which were divided by the knife, recovered.

Not long after the publication of Dr. Blundell's researches, it became known that, as early as the year 1809, Dr. M'Dowal, of Kentucky, had successfully performed one operation for extirpation of a diseased ovary, making an extensive incision into the abdomen, and soon afterwards two nearly similar operations. Mr. Lizars, of Edinburgh, who published these accounts, published at the same time a full narrative of his own unsuccessful attempts in the same direction, coupled, however, with one triumphant case of the major operation, in which he had succeeded in removing a large diseased ovarium. Still the operation excited but little attention in this country, and had never been performed in England, before Dr. Clay, of Manchester, in September last, operated on his first case, nor in London, till November, when the author operated in the case about to be related.

Mrs. F——, æt. 58, applied to Mr. Walne in July last with great abdominal enlargement. The catamenia had ceased four years. Had given birth to five living children, and had several times miscarried. A rounded prominence of the abdomen, circumscribed, with fluctuation, and moveable as a whole, was found on examination: the health was good; no signs of general dropsy. She had observed her gradual increase of size for more than two years; it had, however, given her no pain. Mr. Walne immediately pronounced the case as one of ovarian disease, and, after consulting with Dr. Blundell, determined to extirpate the diseased ovarium by the large abdominal section. Mr. Walne's reasons for choosing the major operation were these—that it does not appear that a less extent of wound diminishes the danger of the operation in any material degree, if at all; and that the complications which occasionally present without being foreseen, and which, indeed, do not admit of being foreseen in every instance, can be more suitably dealt with by the surgeon, through a free opening than through a small one.

On the 6th of November, the operation was performed. The patient was placed on a couch, with her feet upon the ground at its end, and her back supported by pillows. An exploratory incision of the integuments and tendinous expansion, and then of the peritoneum, to the extent of an inch and a half, was first made; a finger was then passed into the peritoneal cavity, and the fluctuating cyst distinguished clearly. The incision was then enlarged from above downwards, to the length of thirteen inches, extending from three inches below the scrobiculus cordis to within one and a half of the pubes, avoiding the umbilicus. The tumor, which proved to be of the right ovary, now advanced gradually through the wound; Mr. Walne then, having given the tumor in charge of an assistant, inserted two fingers behind the broad ligament, and with a needle passed a double ligature behind the pedicle, and thrusting the needle through the middle of that part, brought its point forward. The two halves of the pedicle were then tied separately, and the tumor cut off, immediately in front of the ligature—the enormous mass of more than 16lbs. was now removed, no adhesions whatever interfering. At this period the patient became very sick, and made repeated efforts to vomit, but nothing was brought up. Dr. Freund had been in charge of the divided integuments, and closed them over the abdominal viscera, securing the intestines from exposure to air as much as possible. When the retching efforts had ceased, as there was general oozing rather than any other form of bleeding, it was determined to tie the pedicle in its entire circumference; this was accordingly done; bleeding ceased, and the integuments brought together by about a dozen interrupted sutures. Long pads of lint were laid down each side of the abdomen a little way from the wound, and strips of plaister carried over them from one side of the body to the other.

At the conclusion of the operation the pulse was 76, she was however pale and cold: hot water was applied to the feet, and an anodyne administered. No unfavourable symptom occurred till the second night, when she became hot and restless, and was sick two or three times. On the following evening, she complained of much uneasiness, and suffered from vomiting and occasional eructation of wind. These symptoms were relieved by anodynes. On the following day the sickness had ceased; the tongue was moist and cleaner, and the skin perspiring.

11th. Has not had so good a night; vomiting had twice occurred. On dressing the wound, it was found that the ligatures which had been left out about two inches from the pubic end of the wound, were no longer visible; they had probably been pulled within the wound in one of her fits of restlessness. In the course of the day the tongue became brown and coated, the manner drowsy, and the mind confused; constant nausea, vomiting, hiccup, and pain at the navel; symptoms resembling those of strangulated hernia. These were however relieved by the use of anodynes, enemata, &c.

13th. The wound was dressed, and the remaining sutures removed: adhesive matter covered those parts which were not closed, and which, at three points together, amounted to less than three inches. In the afternoon, she was again seized with symptoms resembling those of incarcerated hernia, and on raising one of the strips of plaister, it was found that one of them had been too tightly applied, lying over a part of the wound not yet quite closed, where intestine was liable to pressure. On removing this piece of plaister, she felt sick and faint, but immediately after was much relieved. This circumstance Mr. Walne considers of importance, as showing that it is not so much peritoneal inflammation, as suffering in the viscera of the abdomen, more particularly the intestines, which is to be apprehended as a consequence of free incision for the removal of diseased ovarium.

From this time she continued rapidly to improve: on the 23rd she sat up for several hours, and on the 29th she felt quite well; the wound was healed, except a small opening at the lower end where the ligatures were lying, and one point by the umbilicus of redundant granulation of the size of half a pea.

The greater portion of the mass removed was fluid, contained in one or more cysts. A substance of about the size of two fists, having at some points a scirrhous hardness and abruptness of form, occupied that part of the tumor where the remains of the fallopian tube, meandering towards its fimbricated extremity sufficiently declared it to be the ovarium of the right side, much enlarged and changed in structure. The fluid is of the ordinary character of ovarian dropsical fluid, and the solid portion is probably of a scirrhous character; the tumor however has not been cut into, Mr. Walne considering that "the disease as nearly in its actual form and size at the period of its removal, as it could be preserved, is more valuable for the surgical illustration of the subject, than when cut into slices for pathological ends, as has been done by hundreds such, to which no other history than that of their fatal influence on the frame that bore them, attaches."

"Note.—The ligatures of the pedicle, which had not come away when the paper went to press, remaining with very little change of position, and being in vain pulled at every other day, on the 6th of January were twisted gently into the form of a cord till resistance was felt, and slight pain excited. They were then fixed in their twisted state by plaister. This proceeding was renewed on the 8th; and on the 10th of January, about ten weeks after the operation, they were drawn out without pain or resistance. In a few days the canal they had occupied closed, and the patient's cure was perfected.

"I was not aware at the time of drawing up the particulars of my own case, that the operation had been performed by Dr. Granville. It appears, however, that that gentleman operated on successfully in 1827, and that he had attempted a similar operation in the previous year, but, on finding extensive adhesions, desisted by the advice of those about him. No professional account of the completed operation was ever published. Of the other I find a brief notice in a medical journal of the period."

*Guilford Street, Russell Square,*
*Jan.* 30, 1843.

---

### THE SYDENHAM SOCIETY.

Prospectus.—The Sydenham Society has been founded for the purpose of meeting certain acknowledged deficiencies in the diffusion of medical literature, which are not likely to be supplied by the efforts of individuals.

It will carry this object into effect by distributing among its members—

1. Reprints of standard English medical works, which are rare and expensive.
2. Miscellaneous Selections from the ancient and from the earlier modern authors, reprinted or translated.
3. Digests of the most important matters contained in old and voluminous authors, British and foreign, with occasional biographical and bibliographical notices.
4. Translations of the Greek and Latin medical authors, and of works in the Arabic and other Eastern languages, accompanied, when it is thought desirable, by the original text.
5. Translations of recent foreign works of merit.
6. Original works of great merit, which might be very valuable as books of reference, but which would not otherwise be published, from not being likely to have a remunerating sale,—such as classified Bibliographies, and alphabetical Indexes to periodical publications and other valuable voluminous works.

The Society will consist of an unlimited number of members.

The subscription constituting a member is one Guinea annually, for which he will be entitled to a copy of every work printed by the Society, during the time of his subscription.

The subscriptions are to be paid in advance; and no member is responsible beyond the amount of his subscription.

All works published by the Society will be selected by the Council; and, previous to publication, will be subjected to their supervision.

The Society will not commence its operations until the number of its members amounts to five hundred.

The works of the Society will be printed for members only; on a uniform plan, and with a good legible type.

The Society will be under the direction of a Council of twenty-four members, elected at the annual general meeting from the subscribers at large; and of this number eighteen only will be re-eligible for the following year.

The President and Vice-Presidents will also be elected annually.

As the expense of management will be very small, nearly the whole of the funds subscribed will be devoted to the publications; and as the proportionate cost of producing books decreases as the number of copies increases, it is anticipated that, when the Society is fully organized, the annual supply of works to members will be considerable.

The great success that has attended other Societies, established on similar principles, and with like objects,—as the Camden, the Parker, the Percy, &c.* leaves no room for doubt as to the eventual prosperity of the Sydenham Society. It would indeed be strange, if Medicine, which boasts of a literature more extensive than that of any other art or science, and of cultivators as numerous, zealous, and learned, as any other department of human knowledge, should fail in attaining an end which has been so speedily and so fully accomplished by the societies referred to, and by others embracing even less comprehensive objects.

As the number of copies printed of any work will be regulated by the number of members, gentlemen desirous of possessing all the Society's publications will see the necessity of giving in their names early.

Gentlemen desirous of becoming members are requested to forward their names to the Provisional Secretary, W. G. Burroughs, Esq. at the Treasurer's, 31, George Street, Hanover Square, or to any member of the Provisional Council.

---

* The present number of subscribers to the Parker Society for publishing theological works, exceeds 7000. The number of members in the Camden is limited to 1200, and that number has long been complete.

## Figures and Tables

**Figure f1:**